# An antisense transcript mediates MALAT1 response in human breast cancer

**DOI:** 10.1186/s12885-019-5962-0

**Published:** 2019-08-05

**Authors:** Carla Pereira Gomes, Sandrina Nóbrega-Pereira, Beatriz Domingues-Silva, Kenny Rebelo, Catarina Alves-Vale, Sérgio Pires Marinho, Tânia Carvalho, Sérgio Dias, Bruno Bernardes de Jesus

**Affiliations:** 1 Instituto de Medicina Molecular, Faculdade de Medicina, Universidade de Lisboa, 1649-028 Lisbon, Portugal; 2 Department of Medical Sciences and Institute of Biomedicine – iBiMED, University of Aveiro, 3810-193 Aveiro, Portugal

**Keywords:** lncRNAs, MALAT1, TALAM1, Migration, Breast cancer

## Abstract

**Background:**

Long non-coding RNAs (lncRNAs) represent a substantial portion of the human transcriptome. LncRNAs present a very stringent cell-type/tissue specificity being potential candidates for therapeutical applications during aging and disease. As example, targeting of MALAT1, a highly conserved lncRNA originally identified in metastatic non-small cell lung cancer, has shown promising results in cancer regression. Nevertheless, the regulation and specificity of MALAT1 have not been directly addressed. Interestingly, MALAT1 locus is spanned by an antisense transcript named TALAM1.

**Methods:**

Here using a collection of breast cancer cells and in vitro and in vivo migration assays we characterized the dynamics of expression and demonstrated that TALAM1 regulates and synergizes with MALAT1 during tumorigenesis.

**Results:**

Down-regulation of TALAM1 was shown to greatly impact on the capacity of breast cancer cells to migrate in vitro or to populate the lungs of immunocompromised mice. Additionally, we demonstrated that TALAM1 cooperates with MALAT1 in the regulation of the properties guiding breast cancer aggressiveness and malignancy.

**Conclusions:**

By characterizing this sense/anti-sense pair we uncovered the complexity of MALAT1 locus regulation, describing new potential candidates for cancer targeting.

**Electronic supplementary material:**

The online version of this article (10.1186/s12885-019-5962-0) contains supplementary material, which is available to authorized users.

## Background

Genome wide studies have assessed that the majority of human and mouse genomes are actively transcribed, resulting in a plethora of coding and non-coding transcripts with potential biological functions [1, 2]. Long non-codifying transcripts (lncRNAs) are the largest class of non-coding transcripts [1]. LncRNAs present, in general, low conservation and a cell type specific expression pattern, guiding to cell-specific functions [3, 4]. Seminal studies on the biology of lncRNAs described their potential function with pathways involved in cancer progression [5] or aging [6]. One of the originally identified cancer-associated lncRNA was MALAT1 (Metastasis-associated lung adenocarcinoma transcript-1) [7, 8]. MALAT1 is a well conserved and widely expressed lncRNA in healthy and malignant tissues [9]. Upregulated expression of MALAT1 is observed in a plethora of tumours including breast, lung, colorectal or cervical cancers [7, 10, 11]. MALAT1 promotes cell motility and is significantly associated with the potential to form metastasis. Additionally, MALAT1 levels are a valuable prognostic for patient survival in tumours of the lung [10]. MALAT1 was shown to be directly involved in pre-mRNA processing, regulation of alternative splicing or control of gene expression through interaction with the polycomb proteins [12]. Little is known, however, about the regulatory and post-processing mechanisms determining MALAT1 cell-type / tissue-specificity. Indeed, MALAT1 is widely expressed in non-cancerous tissues [13]. Post-transcriptionally, the 3′-end of MALAT1 is processed by two RNases (RNase P and RNase Z) generating an uncharacterized small noncoding RNA termed mascRNA that localizes to the cytoplasm [12, 14]. Post-processed MALAT1 transcripts accumulate in different cellular compartments and are very stable due to the formation of tertiary structures [15].

The existence of an antisense transcript co-existing in the MALAT1 locus was firstly observed by Zhao and colleagues. Using RIP-seq they captured and identified PRC2-interacting RNAs in embryonic stem cells where they described an enrichment of an antisense transcript of MALAT1 [16]. Interestingly the sense transcript was not enriched in the PRC2-interacting fraction guiding to independent functions of TALAM1. More recently Zong et al. biochemically described TALAM1, demonstrating their contribution to the stability and transcriptional dynamics of MALAT1 [17]. Despite the biochemical characterization no biological function has been assigned to TALAM1 yet.

Here we investigated the hypothesis whether TALAM1 could be mediating the biological function of MALAT1 during tumorigenesis. Previously, it has been reported that MALAT1 knockdown in breast cancer resulted in alterations in gene expression signatures correlating with differentiation and pro-metastatic signalling [18]. Here we show that TALAM1 synergizes with MALAT1 on the regulation of the migratory capacity of human breast cancer cells. By regulating MALAT1, TALAM1 reveals the functional properties of natural antisense transcripts in gene regulation and cancer networks, raising new candidates for breast cancer targeting.

## Methods

### Cell culture

Breast cancer cell lines (MDA-MB-231 - ATCC® HTB-26™, MCF7 - ATCC® HTB-22™), and the non-tumorigenic cell line MCF10A (gently provided by Sérgio Almeida Lab, IMM-JLA - ATCC® CRL-10317™) were all originally obtained from ATCC. MDA-231-GFP cells were produced from MDA-MB-231 pre-transduced with a lentiviral vector encoding luciferase and GFP, as previously described [19]. Breast cancer cell lines were routinely maintained in DMEM, containing 10% FBS and 2 mM L-Glutamine. MCF10A cells were cultured in DMEM/F12 (Invitrogen) containing 2 mM glutamine and supplemented with 5% heat-inactivated horse serum, epidermal growth factor (20 ng/ml), hydrocortisone (0.5 μg/ml), insulin (10 μg/ml), cholera toxin (1 mg/ml) (all obtained from Sigma). All cell lines were maintained at 37 °C under an atmosphere of 5% CO_2_, tested as being mycoplasma free by using PCR Mycoplasma Test Kit II (AppliChem) and authenticated by examination of morphology and consistent in vitro performance.

### Knockdown experiments

Transient transfection of MCF10A and MDA-MB-231 cells was performed with Lipofectamine RNAiMAX (Invitrogen) following standard procedures. LNA Gapmers for knocking down MALAT1/TALAM1 as well as a non-specific control were designed and purchased from Exiqon and used at a final concentration of 50 mM.

### Strand-specific quantitative real-time PCR (ssRT-qPCR) and subcellular fractionation

Total RNA was extracted with Trizol (Life Technologies) following standard protocols. RNA samples were DNase I treated. For ssRT-qPCR we follow the protocol described in [17], briefly reverse transcription was performed using gene specific reverse transcription primers with a linker sequence at 5′ end, and qPCR was performed using gene specific forward primer and the linker as reverse primer. For subcellular fractionation, we follow previous protocols [20, 21]. Briefly, cells were trypsinized, washed 2 times in PBS and the cell pellet was then resuspended in 1 ml of Lysis Buffer (10 mM Tris-HCl (pH 8–8.4) 0.14 M NaCl, 1.5 mM MgCl2, 0.5% NP-40). The nuclei were collected at centrifuging at 1000 g for 4 min. The supernatant represents the cytoplasmic fraction and was further centrifuged at 11,000 g for 1 min to remove debris. The nuclei were resuspended in 1 ml of Lysis Buffer with 3.3% (w/v) sodium deoxycholate and 6.6% (v/v) Tween 40, centrifuged and washed in lysis buffer. TRIzol was used to extract RNA from the nucleic and cytoplasmic fractions as per manufacturer’s instructions. The RNA samples were DNase I-treated and concentrations determined using a NanoDrop ND1000.

Quantitative real-time PCR was performed in a ViiA™ 7 Real-Time PCR System (Applied Biosystems), using the human primers:

- Actin-For: TGACGTGGACATCCGCAAAG;
- Actin-Rev: CTGGAAGGTGGACAGCGAGG;
- GADPH-For: GACAGTCAGCCGCATCTTCT;
- GADPH-Rev: TTAAAAGCAGCCCTGGTGAC;
- MALAT1-RT1: CGACTGGAGCACGAGGACACTGATTATTTTAATCACCTACAAC;
- MALAT1-RT2: CGACTGGAGCACGAGGACACTGAAGACTGCCAAGTCCTGGAG;
- MALAT1-RT3: CGACTGGAGCACGAGGACACTGATACTCCAAGCATTGGGGAAC;
- MALAT1-RT4: CGACTGGAGCACGAGGACACTGATTCAGGACTCTTTCTGTATTTCTCC;
- TALAM1-RT1: CGACTGGAGCACGAGGACACTGAGGAGTTCTTAAATATCAACCA;
- MALAT1-M1: ATACCAATAGAAGGGCAATG;
- MALAT1-M2: GGAAAGCGAGTGGTTGGTAA;
- MALAT1-M3: GGGTGGGGCTTACTTGTTGT;
- MALAT1-M4: ATGCTGGTGGTTGGCACT;
- TALAM1-L1: GCCCACAGGAACAAGTCCTA;
- Linker-Rev: CGACTGGAGCACGAGGACACTG;
- MIA2-For: AGATTTGTGGGCAGGAAGTAAA;
- MIA2-Rev: CGTTGACATCTGAATTTCCTCA;
- GPC6-For: AGATTATGGCTCTCCGTGTGAT;
- GPC6-Rev: TGTGGTGACAAACTCAAACTCC;
- CDCP1-For: TCAAGATGCAAGAAGGAGTGAA;
- CDCP1-Rev: CGATGATGCACAGACGTTTTAT;

### Droplet digital PCR (ddPCR)

Droplet digital PCR was carried as previously described [22, 23] and briefly detailed hereafter. The ddPCR mixture contained 7,5 μl of 2× ddPCR Evagreen Supermix (Bio-Rad, Hercules, USA), 10 nM of the testing forward and reverse primers or GAPDH primers and 5 ng of cDNA (RNA equivalent) in each 15 μl reaction. The 15 ul reaction was placed in the droplet generator (Bio-Rad #186–3002) resulting in around 20,000 individual droplets [22]. Sealed plates with the droplets were cycled (Veriti DX thermal cycler, ThermoFisher Scientific) under the following conditions: 2 min at 30 °C; 10 min hold at 95 °C; 48 cycles of 95 °C for 50s then 59 °C for 120 s; 5 min at 4 °C, 5 min at 90 °C. After amplification, the plate with the droplets was read on a Bio-Rad droplet reader (QX200) and data was analysed with the Quantasoft software.

### RNA FISH

A set of custom Stellaris® FISH probes designed for MALAT1 and TALAM1 were purchased from Biosearch Technologies. Fish staining was performed according to manufacturer recommendations.

### In vitro cell growth assay

Cells were plated and transfected as previously mentioned. Twenty-four hours after the second transfection, cells from all conditions (Control, α-MALAT1, α-TALAM1 and α-MALAT1 + α-TALAM1) were re-plated at the same cell density into 48-well plates and further incubated for 6 days. The resazurin-based assay was performed at day 0, 2, 4 and 6, by replacing the cell medium with complete medium containing 10% (v/v) resazurin (AlamarBlue) and incubating with cells for 2 h followed by absorbance measurement at 560/590 nm.

### Wound healing assay

MCF10A and MDA-231 cells were seeded into 24-well plates and grown to sub-confluence. Cell proliferation was blocked by a 2 h pre-treatment with MitomycinC (100 ng/ml) in serum-free medium. Afterwards, a scratch was made in each well using a 1000 ul pipette tip and the wounded monolayers washed twice with PBS to remove cell debris and floating cells. Wound width was monitored over time under an inverted microscope with a digital camera. Percentage wound recovery was expressed compared to width of the wound at *t* = 0 (100%).

### In vivo lung colonization assay

Animal experiments were performed according to EU regulations and approved by the Animal Ethics Committee of Instituto de Medicina Molecular (iMM). The animal facility of iMM complies with the Portuguese law for the use of laboratory animals (Decreto-Lei 113/2013); and follows the European Directive 2010/63/EU and the FELASA (Federation of European Laboratory Animal Science Associations) guidelines and recommendations concerning laboratory animal welfare. NSG mice were obtained from the Jackson Laboratories. Animals were healthy throughout the experiment. 36 female mice (ages 5–7 weeks) were injected with MDA-231 breast cancer cells (2 × 10^6^ cells) via tail vein at the animal facility and, 4 weeks later, animals were sacrificed by CO2 and lungs were collected for assessment of lung metastasis by flow cytometry and histological analysis. Animals were randomly divided in 4 groups injected with MDA-231 breast cancer cells (10 controls, 8 α-Malat1, 7 α-Talam1 and 7 α-Malat1/α-Talam1) and 2 groups injected with MDA-231-GFP breast cancer cells (2 controls and 2 MDA-231-GFP/ α-Malat1/α-Talam-1). For flow cytometry determination of CD44-positive cells, briefly, lung tissue (1 g) was digested in a solution containing DNase (10 μg/ml); Collagenase I (0.4 mg/ml, Invitrogen) and Collagenase IV (1 mg/ml, Invitrogen) for 1 h at 37 °C with agitation. Digested lung slurry was strained (40 μm mesh) to a homogenous cell suspension, centrifuged at 300 g for 5 min, followed by Red Cell Lysis using RCLB (Santa Cruz Biotechnology) for 10 min in the dark at room temperature (RT). Cell pellet was re-suspended in PBS and incubated with 10 μl anti-human CD44 Pacific Blue antibody (clone IM7, eBioscience) for 15 min at 4 °C in dark followed by flow cytometry analysis in a BD LSR Fortessa flow cytometer. For histopathology, lungs were harvested, fixed in 10% neutral-buffered formalin, embedded in paraffin and 4 μm sections were stained with haematoxylin and eosin. Tissue sections were examined for screening of metastatic foci by a pathologist blinded to experimental groups in a Leica DM2500 microscope. Representative microphotographs were captured in slides digitally scanned in the Hamamatsu NanoZoomerSQ using the NDPview2 software (Hamamatsu). All mice were euthanized by carbon dioxide asphyxiation at the end of the experiment.

## Results

### Processed MALAT1 and TALAM1 accumulate in HeLa cells

Genome-wide screenings commonly focus on stable and post-processed RNA transcripts. Although these have allowed the characterization of several RNA candidates, it also limited the detection of low expressed or unprocessed RNA species. Recent advance in high sensitive RNA capturing techniques, have permitted an unprecedented visualization of the human genome and its dynamics [24]. To confirm the existence of MALAT1 and TALAM1 we first employed a characterization of the native elongating transcript sequencing (NET-seq), ChromatinRNA and NucleoplasmicRNA of HeLa cells datasets previously published [24]. As previously observed, MALAT1 is highly expressed and their processed and matured isoforms accumulate in HeLa cells [15, 25–27]. Interestingly, NET-seq data allow the detection of the active transcription throughout the entire gene (Additional file 1: Figure S1) [28]. The co-existence of transcripts in the antisense strand of MALAT1 was possible to detect in the Chromatin fraction (Additional file 1: Figure S1, red peaks). Interestingly, TALAM1 accumulates in the region where MALAT1 is cleaved, further associating its expression to the dynamics of MALAT1 processing [17]. Indeed, sense antisense pairs have been show as potential substrates for RNAse P [29].

To further characterize the presence and biological function of TALAM1 we decided to use human representative breast cancer cell lines with different biological and aggressiveness properties, a model known to be regulated by MALAT1 [18, 30]. By presenting different mechanisms of regulation these cells would potentially represent different basal levels of the sense/antisense pair and their isoforms. Correlational studies have previously demonstrated that solely the processed form of MALAT1 could associate with higher mortality in triple-negative breast cancer (Additional file 1: Figure S2, A,C) whereas other regions (such as the mascRNA) do not correlate with tumorigenesis (Additional file 1: Figure S2, B,C). Additionally, the processed isoform is enriched in cancer cell lines (such as the A549 human lung carcinoma cells), whereas the unprocessed isoform could co-exist in non-cancerous tissues (such as in the IMR90 human lung healthy fibroblast line), as evident by the expression patterns depicted in Additional file 1: Figure S3. Overall, differential MALAT1 regulation and processing is therefore supporting its distinct role either promoting cancer growth or co-existing in healthy tissues. Hereafter, we questioned whether TALAM1 may be mediating this specificity and/or shares biological properties with MALAT1.

### TALAM1 correlates with MALAT1 and is upregulated in human breast cancer

**Fig. 1:**
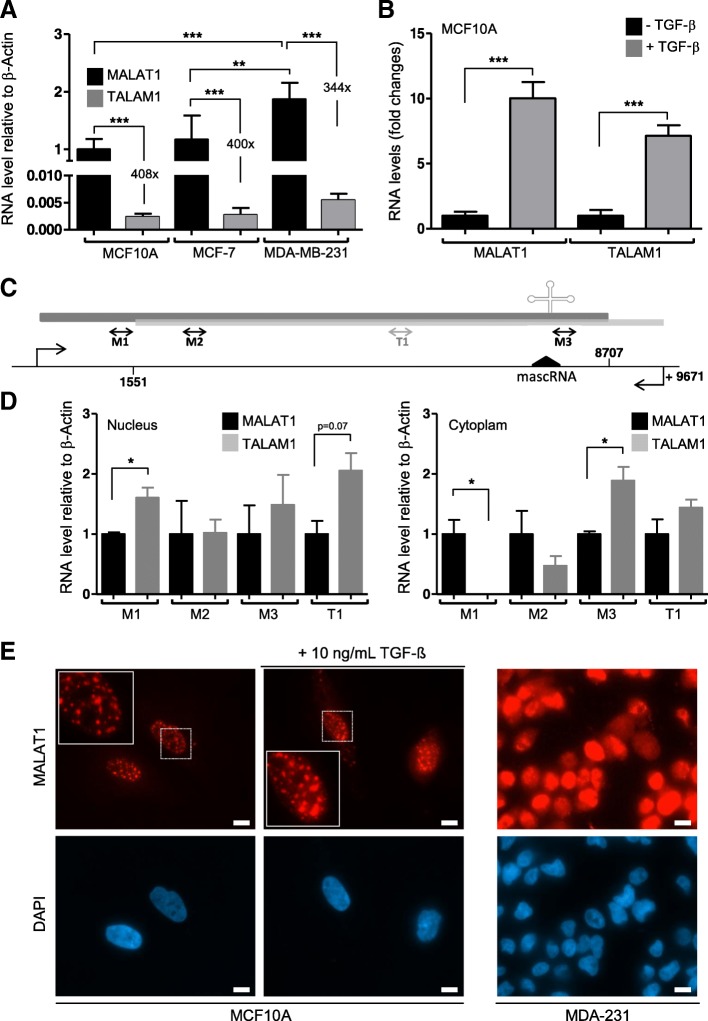
MALAT1 and TALAM1 in breast cancer. **a** Strand-specific RT-qPCR was used for the quantification of MALAT1 and TALAM1 levels in different breast cancer cell lines. MCF10A cells (non-tumorigenic breast cells) were used as control. The results are presented as relative fold-level compared to the MALAT1 levels in MCF10A cells (*n* = 3 ± SD. Two-sided Student’s t-test was used for statistical analysis; ** *p* < 0.01; *** *p* < 0.001). Actin-β was used as reference gene. **b** Strand-specific RT-qPCR was used for the quantification of MALAT1 and TALAM1 levels in MCF10A cells exposed or not to TGF-β (10 ng/ml, for 24 h). The results are presented as relative fold-level compared to the MALAT1 levels in MCF10A cells (*n* = 3 ± SD. Two-sided Student’s t-test was used for statistical analysis; *** *p* < 0.001). Actin-β was used as reference gene. **c** Scheme of the MALAT1/TALAM1 locus with reference for the used primers (dark grey – MALAT1; light grey – TALAM1). **d** Strand-specific RT-qPCR was used for the quantification of MALAT1 and TALAM1 levels in different sub-cellular compartments of MCF10A cells exposed or not to TGF-β (10 ng/ml, for 24 h). The results are presented as relative fold-level compared to the MALAT1 levels in MCF10A cells (*n* = 3 ± SD. Two-sided Student’s t-test was used for statistical analysis; * *p* < 0.05). Actin-β was used as reference gene. **e** Representative images of RNA-FISH in MCF10A cells exposed or not to TGF-β (10 ng/ml, for 24 h) and in MDA-231 cells. Single molecule FISH probes from Stellaris® were used against MALAT1 (red). The corresponding images stained with DAPI are shown (scale bars correspond to 10 μm)

To understand the significance of TALAM1 in human breast cancer, we first determined the expression levels of both MALAT1 and TALAM1 in different human breast cancer cell lines (MCF7 – Luminal A ER^+^/PR^+^, MDA-MB-231 (named hereafter as MDA-231) – Triple negative [mesenchymal features]) [15, 16], and a control non-tumorigenic epithelial breast cell (MCF10A cells). To specifically detect the sense or antisense transcripts, we performed strand specific reverse transcription (ssRT) and the relative levels of both RNAs were quantified by quantitative PCR (Fig. 1a) or droplet digital PCR (ddPCR - Additional file 1: Figure S4) which provides a higher degree of sensitivity and precision [31, 32]. Strand-specific detection of MALAT1 and TALAM1 shown that both lncRNAs are significantly upregulated in all breast cancer cell lines tested comparing to the non-tumorigenic cell line (Fig. 1a and Additional file 1: Figure S4). In accordance with previous publications [17], TALAM1 is 400- to 600-fold less abundant than MALAT1, in the different cell lines tested. The positive correlation detected between MALAT1 and TALAM1 suggests that TALAM1 could share a biological role in the progression of human breast cancer, as reported for MALAT1 [18, 30].

Having the knowledge that MALAT1 was upregulated in cancer tissues, we asked whether non-cancerous tissues would increase MALAT1 / TALAM1 expression after a signal driving migration. Here, MCF10A mammary epithelial cells have been used as a model to investigate epithelial-mesenchymal transition (EMT) pathways in premalignant cells since they are immortal and respond to Transforming Growth Factor β (TGF-β) [33–36]. In particular they progress to a spindle-like morphology losing their tight connections and increasing motility (Additional file 1: Figure S5), as previously observed [37]. MALAT1 was previously shown to be an important mediator of TGF-β induced EMT signalling in bladder cancer cells [38]. Similarly, TGF-β plays an important role in breast cancer tumour invasion and metastasis [39]. After exposing MCF10A mammary epithelial cells to TGF-β (Additional file 1: Figure S5) a general increase in both sense and antisense lncRNAs was observed (Fig. 1b). To determine the cellular localization of both transcripts after TGF-β cellular fractionation was performed. An increase in both lncRNAs was detected in the nucleus (non-chromatin binding, Fig. 1c,d) whether the processed form increased in the cytoplasm (Fig. 1d). To confirm the cellular localization of MALAT1, RNA-fluorescent in situ hybridization (RNA-FISH) was performed in the non-tumorigenic MCF10A cell line using *Stellaris* probes specifically designed for MALAT1 (Fig. 1e). MALAT1 accumulates preferentially in the nuclear speckles as previously described [17]. After exposing MCF10A to TGF-β, MALAT1 adopts a more homogenous distribution in the nucleus, although less expressed but guiding to the distribution observed in MDA-231 cells (Fig. 1e). Of noteworthy, TALAM1 could not be detected in the nucleus with *Stellaris* probes in the presence or absence of TGF-β (Additional file 1: Figure S6). TALAM1 probes stained preferentially the cytoplasm. Although TALAM1 signal could probably shuffle between the cytoplasm and the nucleus, further experimentation will be needed to fully support the presence of TALAM1 in the cytoplasm.

### TALAM1 synergizes with MALAT1 in the regulation of cell growth and mobility

**Fig. 2:**
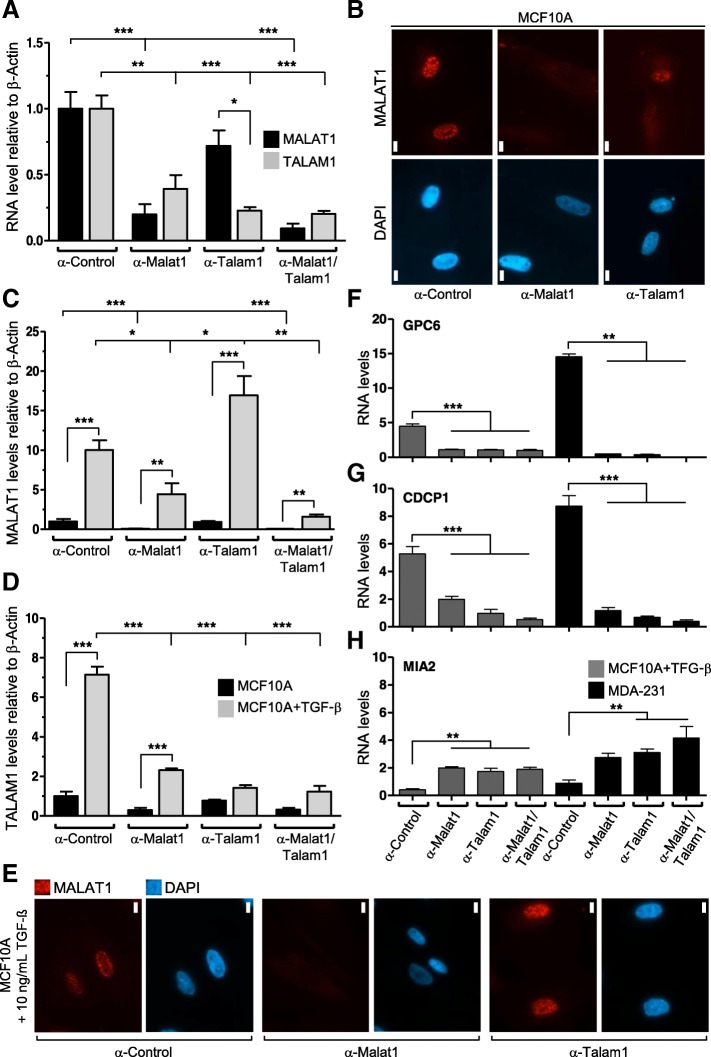
MALAT1 and TALAM1 regulate the metastatic potential of triple negative breast cancer cells. **a** Quantification of MALAT1 and TALAM1 expression levels by ssRT-qPCR after incubation with specific antisense oligonucleotides (LNA Gapmers, Exiqon) in MCF10A cells without TGF-β (*n* = 3 ± SD. Two-sided Student’s t-test was used for statistical analysis; * *p* < 0.05; ** *p* < 0.01). Actin-β was used as reference genes. **b** Representative images of RNA-FISH in MCF10A cells after the indicated treatments. Single molecule FISH probes from Stellaris® were used against MALAT1 (red). The corresponding images stained with DAPI are shown (scale bars correspond to 10 μm). **c**, **d**) Quantification of MALAT1 (**c**) and TALAM1 (**d**) expression levels by ssRT-qPCR after incubation with specific antisense oligonucleotides (LNA Gapmers, Exiqon) in MCF10A cells exposed to 10 ng/ml of TGF-β (*n* = 3 ± SD. Two-sided Student’s t-test was used for statistical analysis; *p < 0.05; **p < 0.01; ***p < 0.001). Actin-β was used as reference genes. **e** Representative images of RNA-FISH in MCF10A cells exposed to TGF-β after the indicated treatments. Single molecule FISH probes from Stellaris® were used against MALAT1 (red). The corresponding images stained with DAPI are shown (scale bars correspond to 10 μm). **f**-**h** Quantification of the levels of the metastatic markers GPC8 (**f**), CDCP1 (**g**) and MIA2 (**h**) by qPCR after incubation with specific antisense oligonucleotides (LNA Gapmers, Exiqon) in MCF10A cells exposed to TGF-β and in the triple-negative MDA-231 breast cancer cell line (*n* = 3 ± SD. Two-sided Student’s t-test was used for statistical analysis; **p < 0.01). Actin-β was used as reference genes

Given the detection of the highest expression levels of TALAM1 in the triple negative breast cancer cell line MDA-MB-231 (Fig. 1a) as well as in epithelial breast cells exposed to TGF-β (Fig. 1b), we next enquired about the biological role of this natural antisense transcript (NAT) in the functional properties of breast cancer cells. To test the specific roles of the sense and antisense transcripts we used locked nucleic acids (LNAs) [40] against MALAT1 and TALAM1 either separately or in combination and a non-specific antisense oligonucleotide as control. LNA against MALAT1 (referred hereafter as α-MALAT1) resulted in a 80% decrease of MALAT1 levels and 60% of TALAM1, while cells transfected with LNA against TALAM1 (α-TALAM1) lead to a knockdown of 80% of TALAM1 and 30% of MALAT1 (Fig. 2a,b). When combining both LNAs, a downregulation of 90% of the sense transcript was achieved, while the antisense levels remained similar to those obtained with α-TALAM1 LNA (Fig. 2a). Additionally, downregulation of MALAT1 and TALAM1 was similarly achieved in MCF10A cells exposed to TGF-β (Fig. 2c,d). Interestingly, downregulation of TALAM1 lead to higher levels of MALAT1 after exposure to TFGβ in MCF10A cells (Fig. 2c,e), overall suggesting that TALAM1 could be mediating the biological response of MALAT1 after exposure to TGF-β by controlling the processed and active forms of MALAT1.

To investigate the response to lower levels of MALAT1 and TALAM1 we focused on the alterations of signalling pathways involved in the regulation of invasion and metastasis. Different metastatic-associated gene targets were shown to be altered after MALAT1 deregulation [10]. Down-regulation of MALAT1 or TALAM1 expression induced alterations in the pro-metastatic signature of MDA-231 or MCF10A exposed to TGF-β including the genes *GPC6*, *CDCP1* and *MIA2*. Interestingly, downregulation of TALAM1 alone gave similar, or superior results, comparing to the downregulation of MALAT1 (Fig. 2f-h).

### TALAM1 down-regulation significantly impairs mobilization of human breast cancer cells

**Fig. 3:**
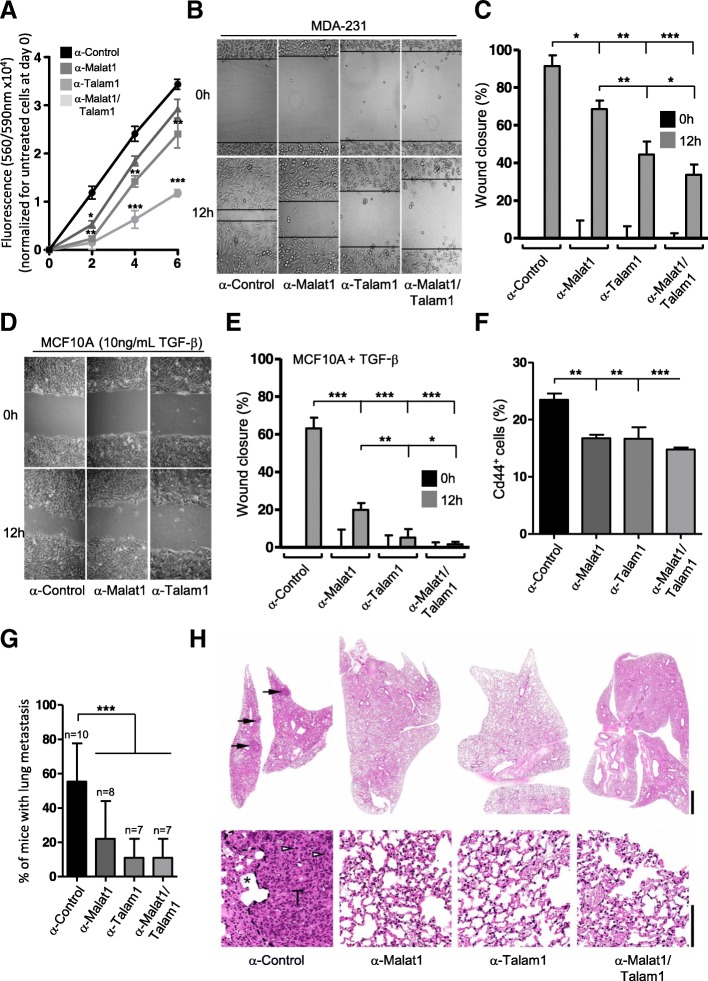
TALAM1 synergizes with MALAT1 in breast cancer cell migration and invasion. **a** Quantification of MDA-MB-231 cells proliferative capacity up to 6 days after specific knockdown of MALAT1 or/and TALAM1 levels by determination of cellular metabolic activity through the resazurin-based assay (*n* = 3 ± SD. Two-sided Student’s t-test was used for statistical analysis; * *p* < 0.05; ** *p* < 0.01; *** *p* < 0.001). **b** Representative images of the wound healing assay performed in MDA-MB-231 cells after the specific knockdown of MALAT1 or/and TALAM1. **c** Percentage of wound closure quantified from the images acquired in the wound healing assay in (B) MDA-MB-231 cells (*n* = 3 ± SD. Two-sided Student’s t-test was used for statistical analysis; * *p* < 0.05; ** *p* < 0.01; *** *p* < 0.001). **d** Representative images of the wound healing assay performed in MCF10A cells exposed to 10 ng/ml of TGF-β after the specific knockdown of MALAT1 or/and TALAM1. **e** Percentage of wound closure quantified from the images acquired in the wound healing assay in (C) MDA-MB-231 cells (*n* = 3 ± SD. Two-sided Student’s t-test was used for statistical analysis; * *p* < 0.05; ** *p* < 0.01; *** *p* < 0.001). **f** Flow cytometry determination of CD44-positive cells from lung tissue (1 g) of mice injected with MDA-MB-231 cells downregulated for the MALAT1, TALAM1 or both. Cells treated with a control LNA were used as a control. (*n* = 3 ± SD. Two-sided Student’s t-test was used for statistical analysis; ** *p* < 0.01; *** *p* < 0.001) **g**) Percentage of mice presenting metastatic lesions after tail vein injection of MDA-MB-231 cells treated with the labelled conditions. Metastatic foci were assessed by an authorized pathologist and were composed of tumour cells with a solid pattern, with frequent mitosis. Number of mice per group is above the bars (Chi-squared test was used for statistical analysis; *** *p* < 0.001). **h** Representative microphotographs of the lung of mice injected with MDA-231 cells treated for 48 h with the labelled LNAs. Tumour metastasis (black arrow) was majorly seen with MDA-MB-231 cells treated with the control LNA. Metastatic foci (inside dashed line) expanded the lung (*, alveoli) and were composed of tumour cells with a solid pattern and high mitotic index (white arrowhead). *Hematoxylin and Eosin. Scale bar 1 mm (upper panel) and 100um (lower panel)*

The migratory and proliferative potentials of MDA-231 or MCF10A + TGF-β cells are an indirect measurement of their aggressive potential [41]. To measure the impact of α-MALAT1 or α-TALAM1 separately or together on cell proliferation, we employed LNAs with a resazurin-based assay during a time course of 6 days (0, 2, 4 and 6 days). The transfection with the scrambled control did not alter the cellular proliferative capacity, which presents a continuous and linear growth along the six days in culture. In contrast, the incubation with α-MALAT1 LNAs resulted in a decreased rate of growth in the first days (Fig. 3a) although in the following days no significance differences were observed. The downregulation of TALAM1 resulted in a more pronounced effect in cell growth arrest observed in all the time-points tested. Remarkably, the combinatorial downregulation of both transcripts resulted in a synergistic effect, presenting a 4-fold decrease in cell growth and proliferative capacity comparing to the control condition (Fig. 3a).

To infer about the role of TALAM1 in the migratory capacity of cells, both MDA-231 and MCF10A (in the presence of 10 ng/μl of TGF-β) were subjected to the wound healing assay in the presence of MitomycinC, abolishing cell division. Down-regulation of MALAT1 leads to a reduction of the migratory capacity of MDA-231 and MCF10A + TGF-β cells (Fig. 3b-e, in the absence of TGF-β MCF10A capacity to migrate is abolished (data not shown)), in agreement with previous reports in other cancer models [10, 42]. Although wound widths in α-MALAT1 conditions were notably larger than in those transfected with control LNA at 12 h, the wound eventually closed at 24 h. Down-regulation of TALAM1 and, in particular, the concomitant downregulation of both transcripts lead to a stronger effect as observed by the impaired migration capacity, and wound preservation (Fig. 3b-e). This result supports the biological function of TALAM1, at least when concomitantly targeted with MALAT1.

Next, we assessed whether MALAT1 and TALAM1 down-regulation would impact the ability of MDA-231 cells to colonize the lungs of NSG mice upon IV injection. NSG mice were injected with 2 × 10^6^ of MDA-231 cells that were either targeted for MALAT1, TALAM1 or both (all assays compared with MDA-231 cells treated with a control LNA). Four weeks later, the mice were sacrificed and the lungs were collected to assess the development of lung metastasis. Quantification of CD44^+^ cells by flow cytometry demonstrates that downregulation of MALAT1, TALAM1 and, more robustly when both transcripts were targeted, strongly affects the capacity of human breast cancer cells to colonize the lungs of immuno compromised mice, as displayed by the percentage of lung CD44^+^ cells measured by flow cytometry (Fig. 3f and Additional file 1: Figure S7). CD44 is a trans-membrane glycoprotein involved in several cellular processes, including migration and adhesion [43] and is widely used as a surface marker to isolate cancer stem cells from solid tumours [44]. Histological examination revealed more undifferentiated lung metastasis in the control group comparing to mice that were targeted for MALAT1, TALAM1 and both transcripts (Fig. 3e,f). Additionally, NSG mice were injected with 2 × 10^6^ of MDA-231-GFP cells that were either targeted for both MALAT1/TALAM1 transcripts or with a control LNA, and only the control cells were able to accumulate in the lungs of NSG mice (Additional file 1: Figure S8). Overall, these results support a biological role for TALAM1 in breast cancer progression and discriminate a potential novel target stalling cancer invasiveness.

## Discussion

Natural antisense transcripts, occurring in the antisense strand of coding or non-coding genes, are common RNA species in the human and mouse genomes [2] still, only few have biological roles assigned [45]. Interestingly, a new study describes the role of a sense-antisense node in neuroblastoma susceptibility [46].

Here, we focused on TALAM1, an antisense transcript co-existing on the MALAT1 locus. The existence of a NAT was previously described by Zhao and colleagues using RIP-seq [16, 17]. Interestingly, they observed that TALAM1 could bind PRC2 complex, something not detected with the sense transcript. Although TALAM1 was later identified has regulating the functional levels of MALAT1 in *cis*, PRC2 specific-binding capacity may be mediating other non-canonical functions for TALAM1 [17].

By regulating the functional levels of MALAT1, down-regulation of TALAM1 leads to a synergistic biological effect characterized by a stronger decrease of the migration, invasion and expression properties of human breast cancer cells. In particular, in the presence of TGF-β, TALAM1 regulates the processed form of MALAT1 whether absence of TALAM1 in this scenario results in an uncontrolled expression and accumulation of MALAT1. Although not assessed here, the binding of TALAM1 to the PRC2 complex may be mediating the response of MALAT1 to TGF-β. Indeed, suz12 (a core component of the polycomb repressive complex 2) knockdown inhibits tumour metastasis in vivo via MALAT1 [47]. However, we cannot rule out other biological functions in *trans* for TALAM1, a possibility that is currently being addressed.

## Conclusion

Regulation of MALAT1 through a NAT represents the complexity of the human genome and, in particular, the biological relevance of non-coding antisense transcripts regulating both coding and non-coding genes. Here, we demonstrate that TALAM1 synergizes with MALAT1 during tumorigenesis in aggressive breast cancer. In particular our study provides evidence for the biological relevance of non-coding antisense transcripts during cancer progression, describing new potential candidates for cancer targeting.

## Additional file

Additional file 1: Supplementary figures. (PPT 1919 kb)

## Data Availability

All data generated or analyzed during this study are included in this published article and its additional files.
