# Supplementary material for: An antisense transcript mediates MALAT1 response in human breast cancer
Source: BMC Cancer. 2019 Aug 5;19:771. doi: 10.1186/s12885-019-5962-0 (PMC6683341; doi:10.1186/s12885-019-5962-0)
Supplement: Supplementary file 1 — Supplementary figures. (PPT 1919 kb) [file 12885_2019_5962_MOESM1_ESM.ppt]

## Slide 1
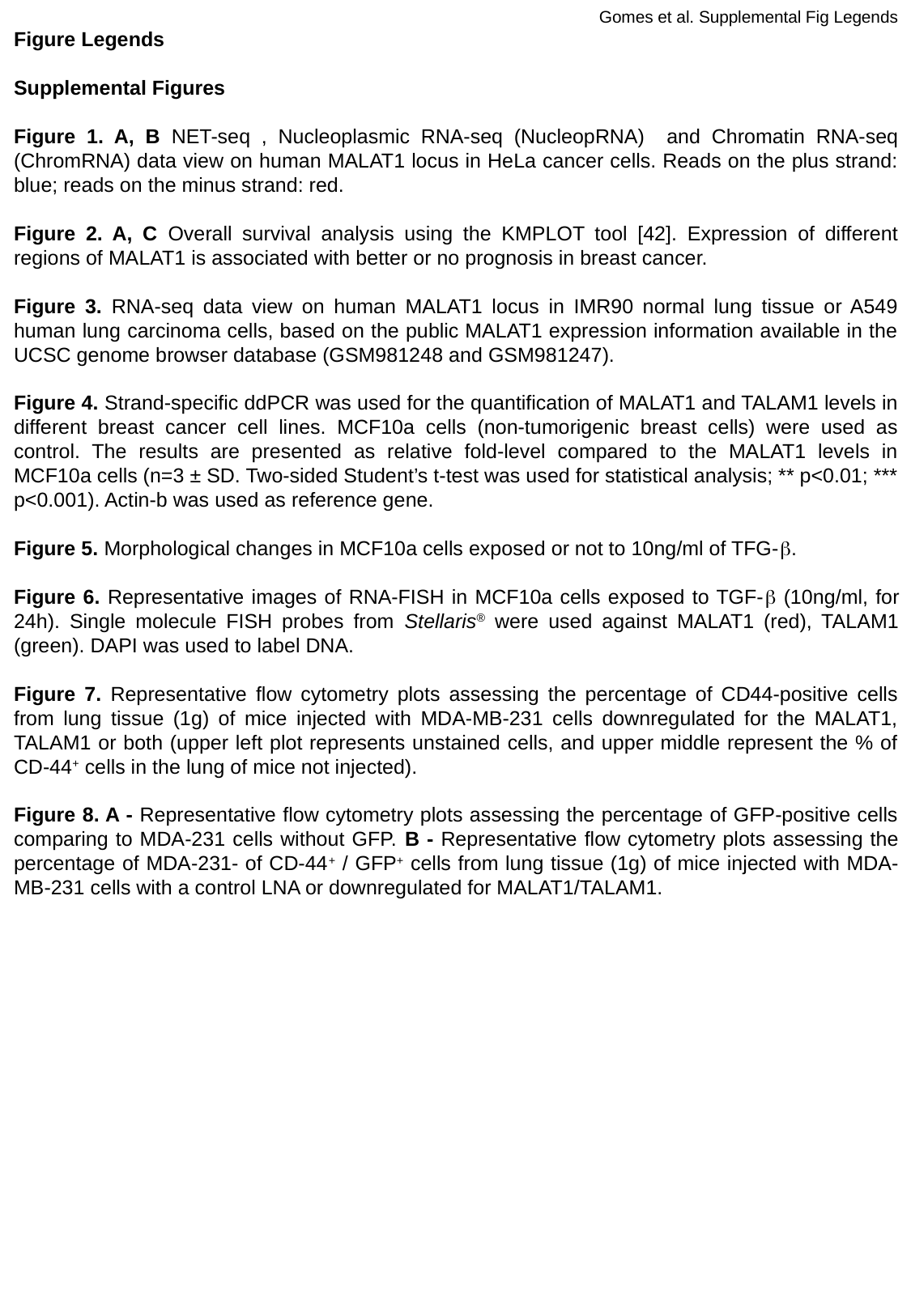

Gomes et al. Supplemental Fig Legends
Figure Legends
Supplemental Figures
Figure 1. A, B NET-seq , Nucleoplasmic RNA-seq (NucleopRNA) and Chromatin RNA-seq (ChromRNA) data view on human MALAT1 locus in HeLa cancer cells. Reads on the plus strand: blue; reads on the minus strand: red.
Figure 2. A, C Overall survival analysis using the KMPLOT tool [42]. Expression of different regions of MALAT1 is associated with better or no prognosis in breast cancer.
Figure 3. RNA-seq data view on human MALAT1 locus in IMR90 normal lung tissue or A549 human lung carcinoma cells, based on the public MALAT1 expression information available in the UCSC genome browser database (GSM981248 and GSM981247).
Figure 4. Strand-specific ddPCR was used for the quantification of MALAT1 and TALAM1 levels in different breast cancer cell lines. MCF10a cells (non-tumorigenic breast cells) were used as control. The results are presented as relative fold-level compared to the MALAT1 levels in MCF10a cells (n=3 ± SD. Two-sided Student’s t-test was used for statistical analysis; ** p<0.01; *** p<0.001). Actin-b was used as reference gene.
Figure 5. Morphological changes in MCF10a cells exposed or not to 10ng/ml of TFG-β.
Figure 6. Representative images of RNA-FISH in MCF10a cells exposed to TGF-β (10ng/ml, for 24h). Single molecule FISH probes from Stellaris® were used against MALAT1 (red), TALAM1 (green). DAPI was used to label DNA.
Figure 7. Representative flow cytometry plots assessing the percentage of CD44-positive cells from lung tissue (1g) of mice injected with MDA-MB-231 cells downregulated for the MALAT1, TALAM1 or both (upper left plot represents unstained cells, and upper middle represent the % of CD-44+ cells in the lung of mice not injected).
Figure 8. A - Representative flow cytometry plots assessing the percentage of GFP-positive cells comparing to MDA-231 cells without GFP. B - Representative flow cytometry plots assessing the percentage of MDA-231- of CD-44+ / GFP+ cells from lung tissue (1g) of mice injected with MDA-MB-231 cells with a control LNA or downregulated for MALAT1/TALAM1.

## Slide 2
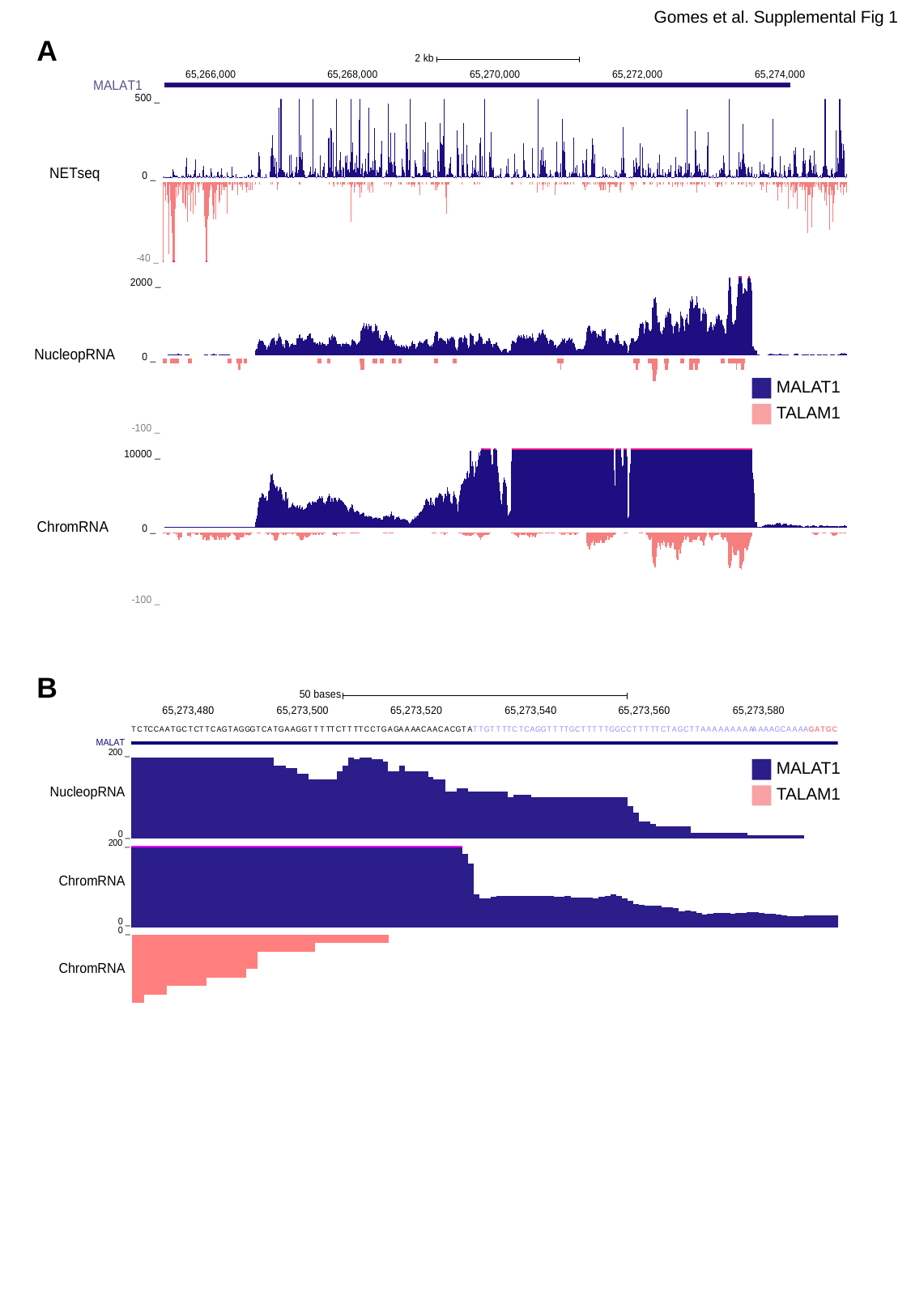

Gomes et al. Supplemental Fig 1
A
MALAT1
TALAM1
B
MALAT1
TALAM1

## Slide 3
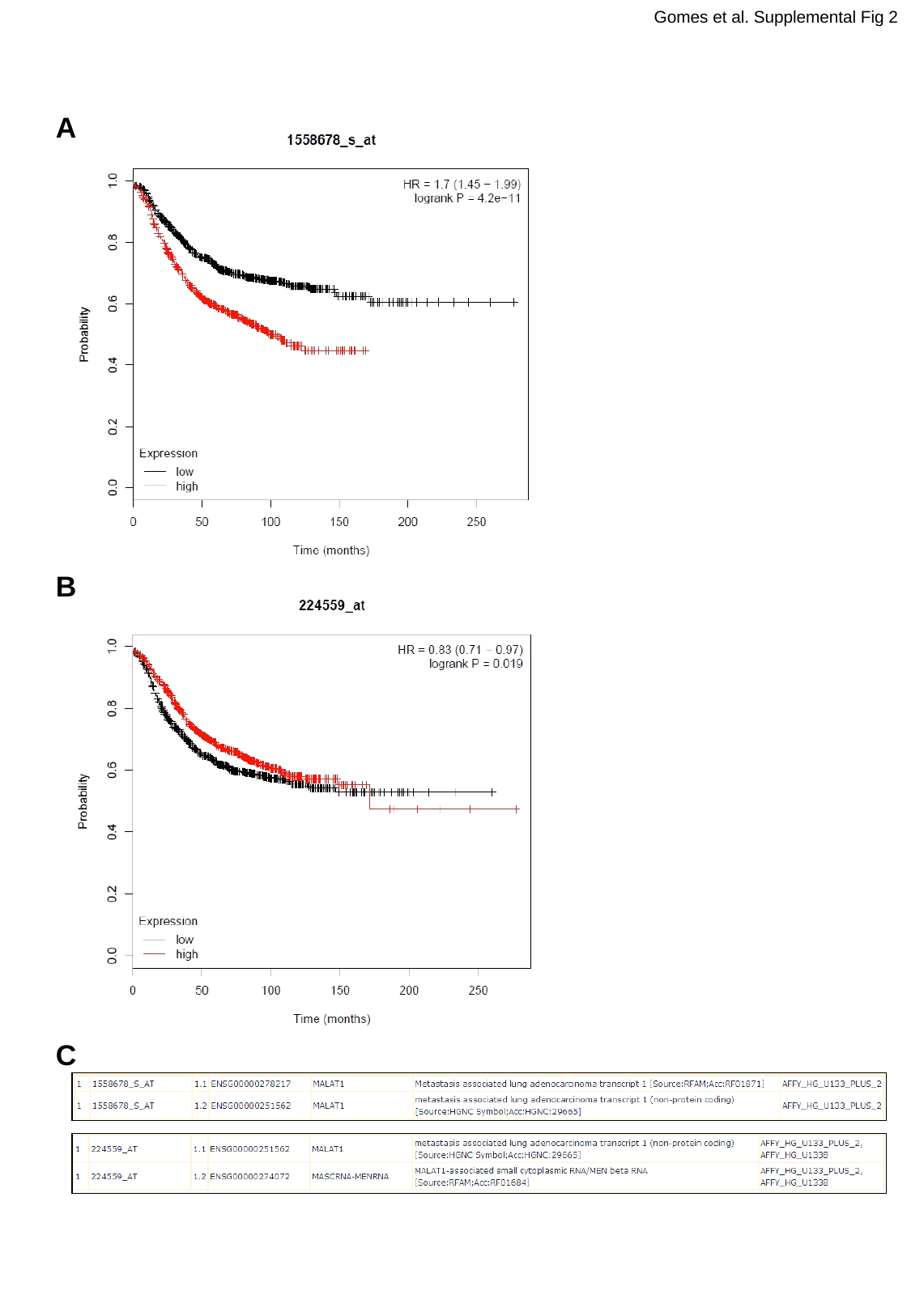

Gomes et al. Supplemental Fig 2
A
B
C

## Slide 4
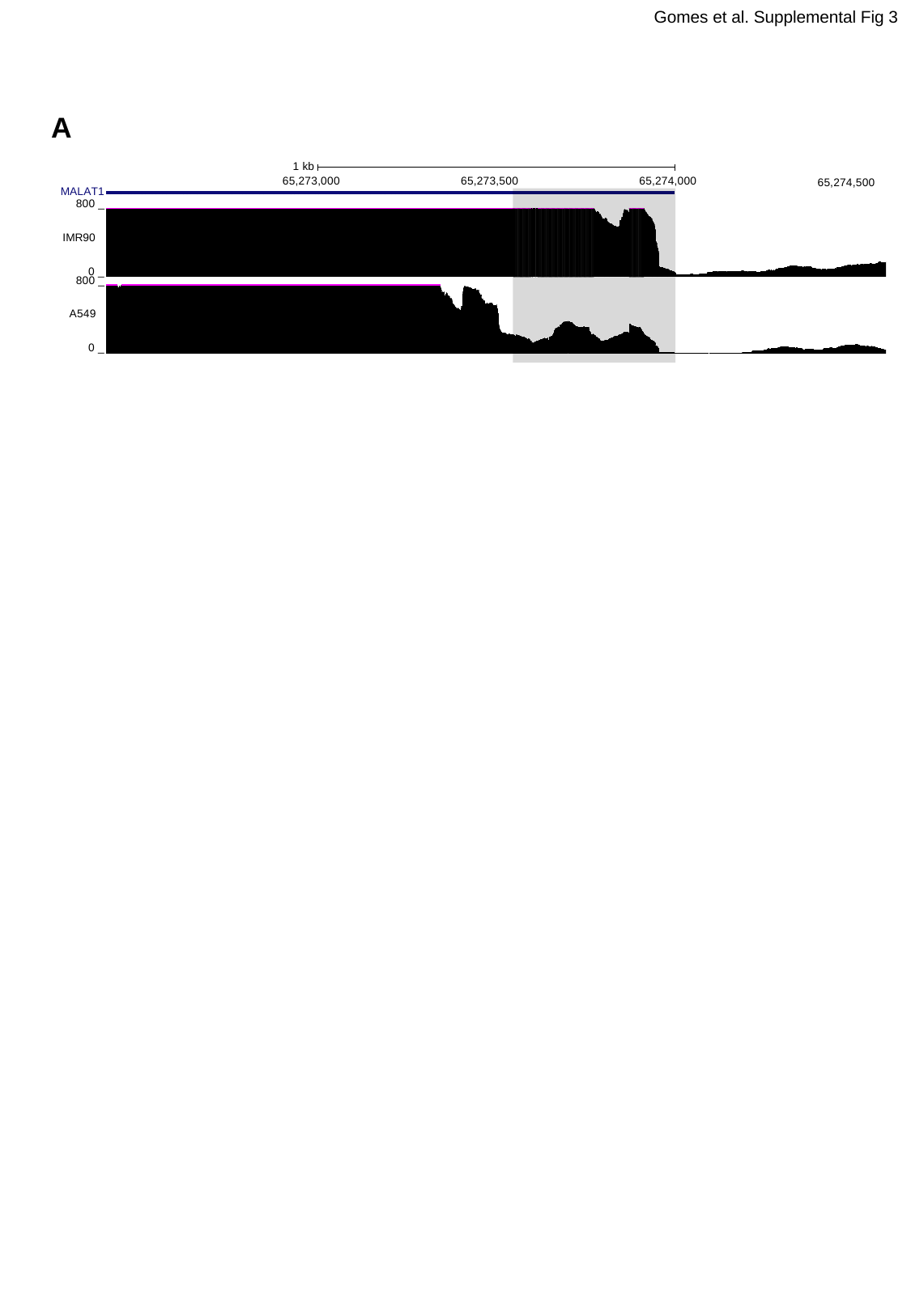

Gomes et al. Supplemental Fig 3
A

## Slide 5
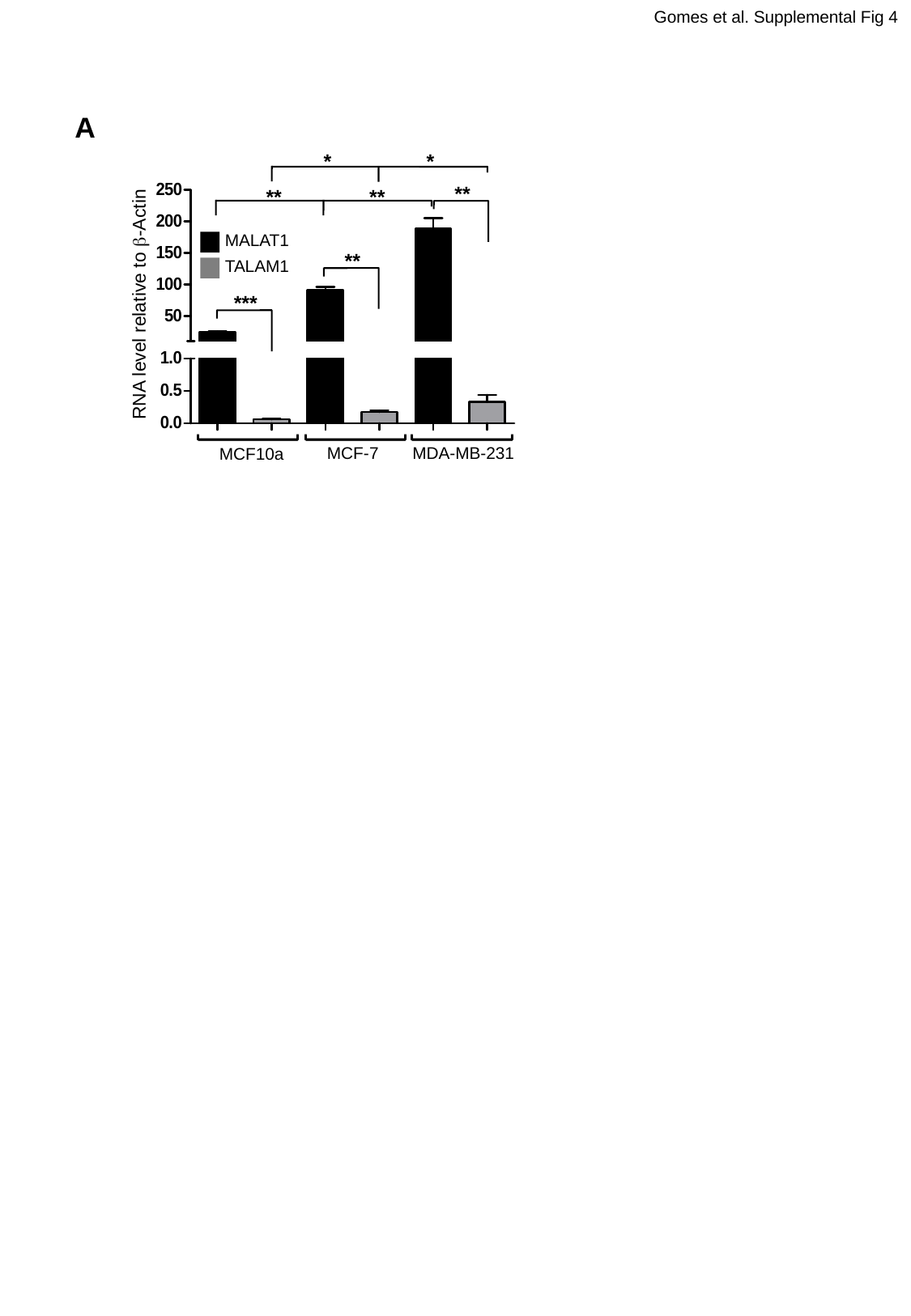

Gomes et al. Supplemental Fig 4
A
*
*
**
**
**
MALAT1
**
TALAM1
***
RNA level relative to β-Actin
MCF-7
MDA-MB-231
MCF10a

## Slide 6
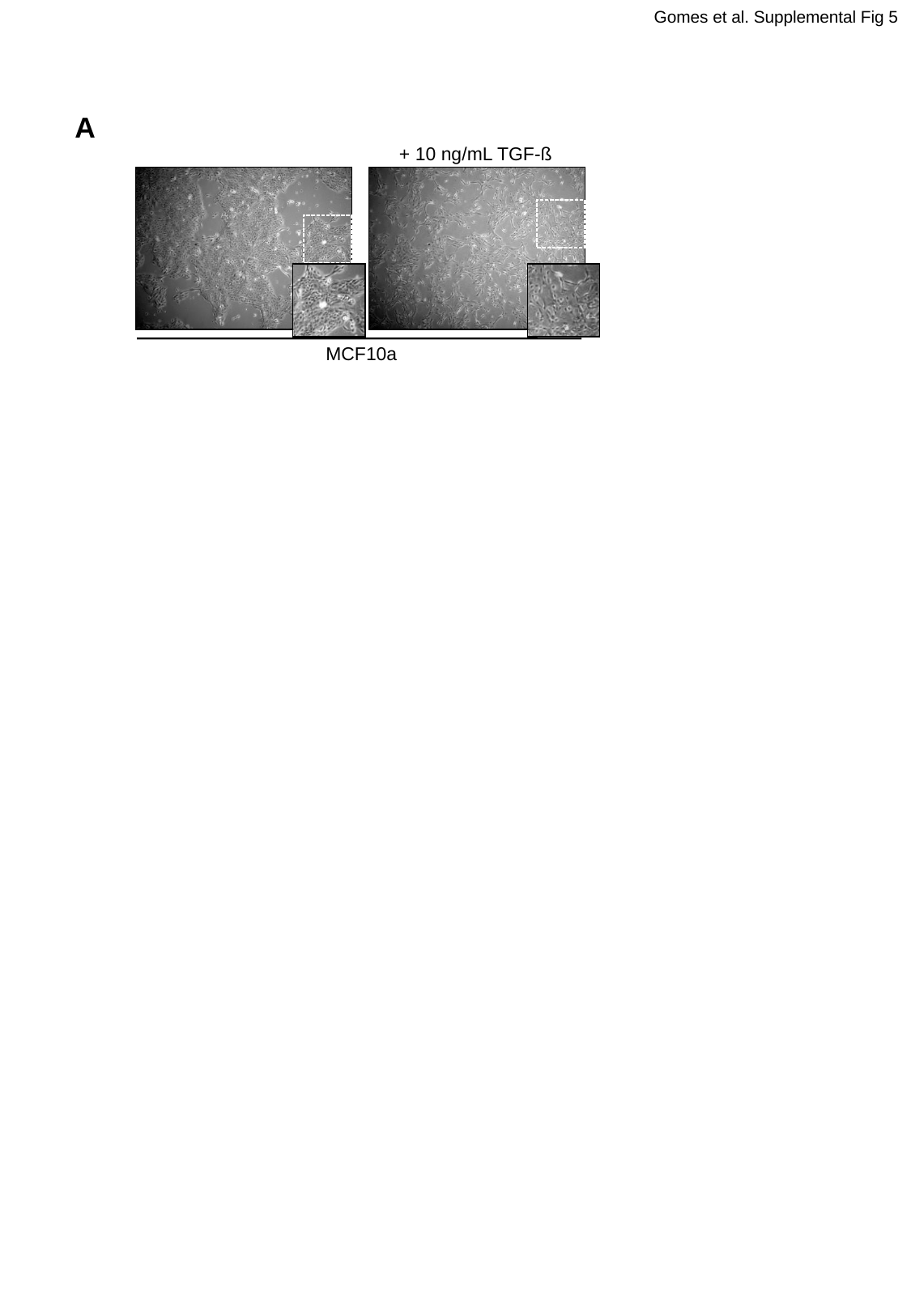

Gomes et al. Supplemental Fig 5
A
+ 10 ng/mL TGF-ß
MCF10a

## Slide 7
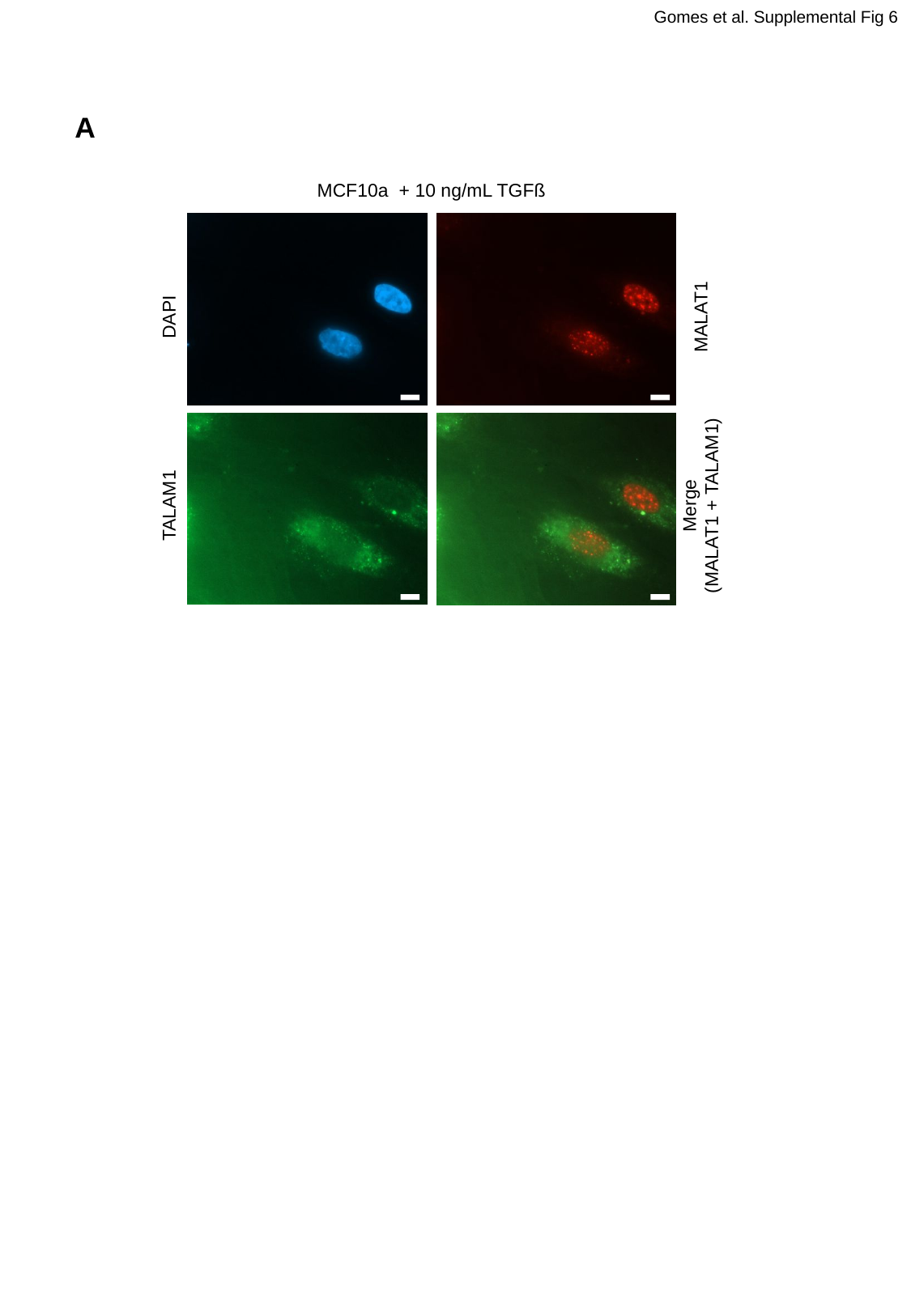

Gomes et al. Supplemental Fig 6
A
MCF10a + 10 ng/mL TGFß
MALAT1
DAPI
Merge
(MALAT1 + TALAM1)
TALAM1

## Slide 8
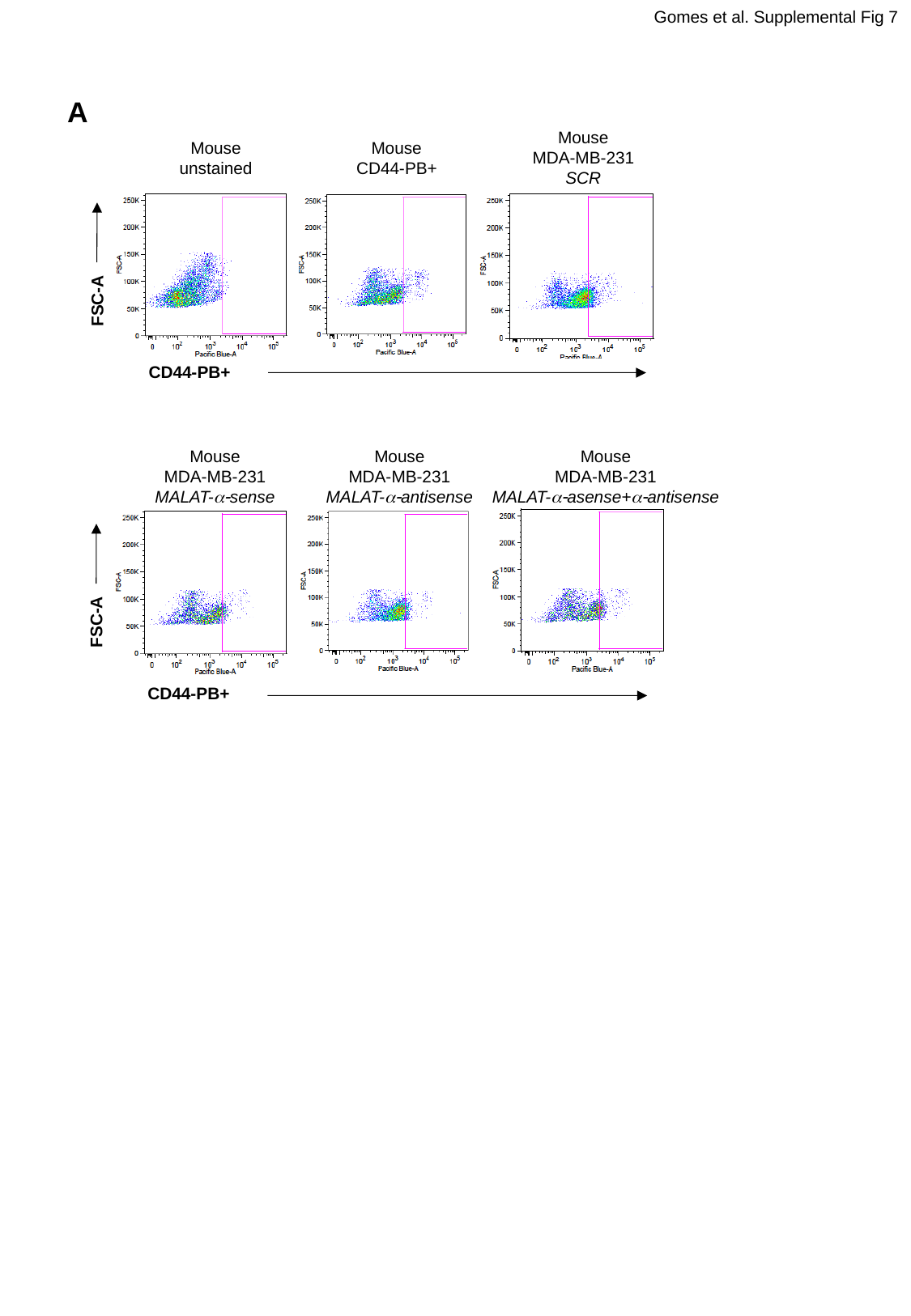

Gomes et al. Supplemental Fig 7
A
Mouse
MDA-MB-231
SCR
Mouse
unstained
Mouse
CD44-PB+
FSC-A
CD44-PB+
Mouse
MDA-MB-231
MALAT-α−sense
Mouse
MDA-MB-231
MALAT-α−antisense
Mouse
MDA-MB-231
MALAT-α−asense+α−antisense
FSC-A
CD44-PB+

## Slide 9
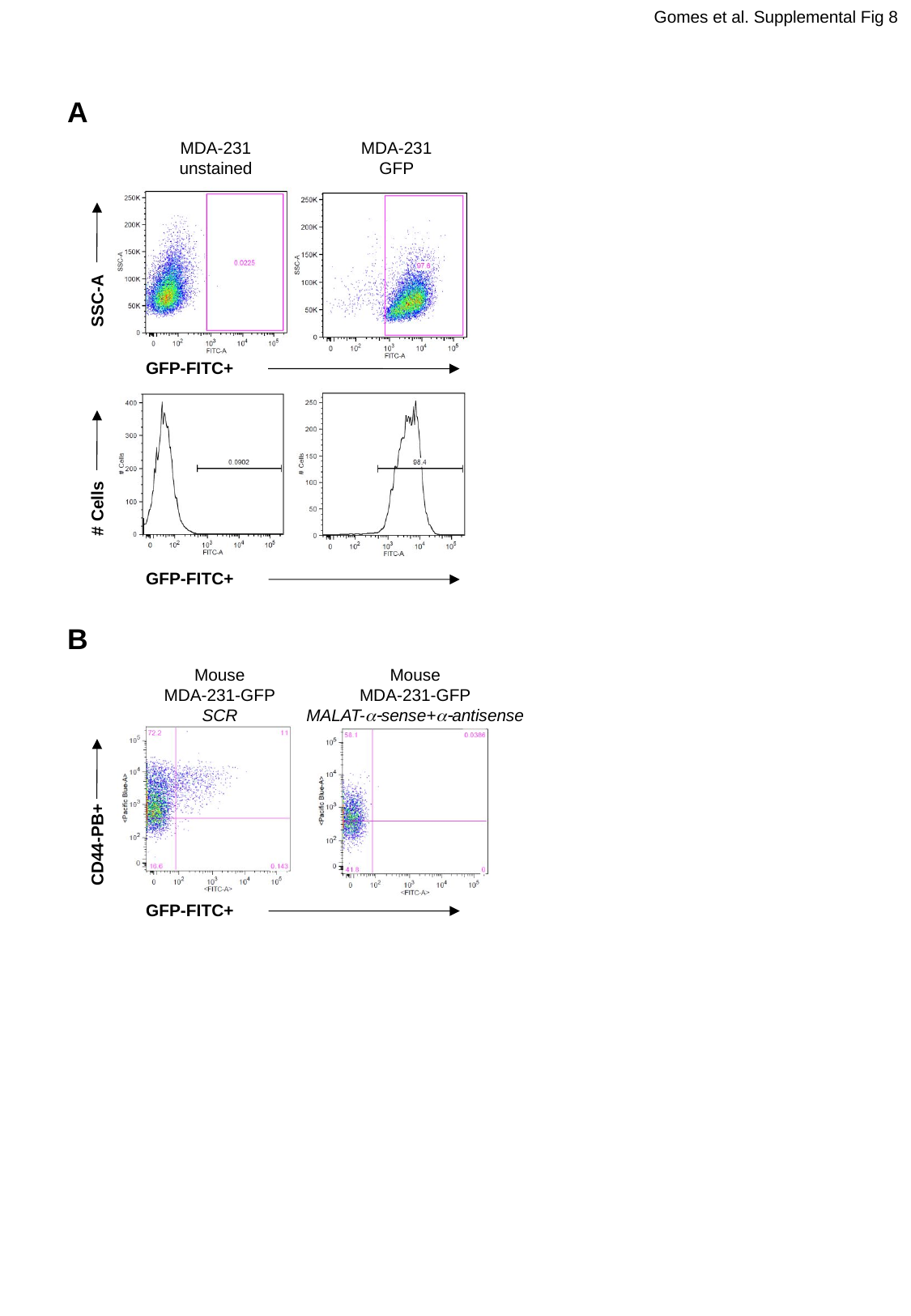

Gomes et al. Supplemental Fig 8
A
MDA-231
unstained
MDA-231
GFP
SSC-A
GFP-FITC+
# Cells
GFP-FITC+
B
Mouse
MDA-231-GFP
SCR
Mouse
MDA-231-GFP
MALAT-α−sense+α−antisense
CD44-PB+
GFP-FITC+
